# Using machine learning to extract information and predict outcomes from reports of randomised trials of smoking cessation interventions in the Human Behaviour-Change Project

**DOI:** 10.12688/wellcomeopenres.20000.1

**Published:** 2023-10-12

**Authors:** Robert West, Francesca Bonin, James Thomas, Alison J. Wright, Pol Mac Aonghusa, Martin Gleize, Yufang Hou, Alison O'Mara-Eves, Janna Hastings, Marie Johnston, Susan Michie

**Affiliations:** 1 Research Department of Behavioural Science and Health, University College London, London, England, UK; 2 IBM Research Europe, Dublin, Ireland; 3 EPPI-Centre, Social Research Institute, University College London, London, England, UK; 4 Institute of Pharmaceutical Science, King's College London, London, England, UK; 5 Institute for Implementation Science in Health Care, Faculty of Medicine, University of Zurich, Zürich, Zurich, Switzerland; 6 School of Medicine, University of St Gallen, St. Gallen, St. Gallen, Switzerland; 7 Aberdeen Health Psychology Group, University of Aberdeen, Aberdeen, Scotland, UK; 8 Centre for Behaviour Change, University College London, London, England, UK

**Keywords:** behaviour change interventions, artificial intelligence, machine learning, natural language processing, prediction systems, information extractions, ontologies, evidence synthesis

## Abstract

**Background**

Using reports of randomised trials of smoking cessation interventions as a test case, this study aimed to develop and evaluate machine learning (ML) algorithms for extracting information from study reports and predicting outcomes as part of the Human Behaviour-Change Project. It is the first of two linked papers, with the second paper reporting on further development of a prediction system.

**Methods**

Researchers manually annotated 70 items of information (‘entities’) in 512 reports of randomised trials of smoking cessation interventions covering intervention content and delivery, population, setting, outcome and study methodology using the Behaviour Change Intervention Ontology. These entities were used to train ML algorithms to extract the information automatically. The information extraction ML algorithm involved a named-entity recognition system using the ‘FLAIR’ framework. The manually annotated intervention, population, setting and study entities were used to develop a deep-learning algorithm using multiple layers of long-short-term-memory (LSTM) components to predict smoking cessation outcomes.

**Results**

The F1 evaluation score, derived from the false positive and false negative rates (range 0–1), for the information extraction algorithm averaged 0.42 across different types of entity (SD=0.22, range 0.05–0.88) compared with an average human annotator’s score of 0.75 (SD=0.15, range 0.38–1.00). The algorithm for assigning entities to study arms ( *e.g.*, intervention or control) was not successful. This initial ML outcome prediction algorithm did not outperform prediction based just on the mean outcome value or a linear regression model.

**Conclusions**

While some success was achieved in using ML to extract information from reports of randomised trials of smoking cessation interventions, we identified major challenges that could be addressed by greater standardisation in the way that studies are reported. Outcome prediction from smoking cessation studies may benefit from development of novel algorithms, *e.g.*, using ontological information to inform ML (as reported in the linked paper ^3^).

## Introduction

Changing human behaviour at scale is necessary to address many of the challenges facing humankind ^1^. Behavioural science aims to discover better ways of achieving this. Much of the research involves using randomised controlled trials to evaluate behaviour change interventions (BCIs) (see Appendix 1 in *Extended data* for a glossary of terms and abbreviations). The results of these trials need to be synthesised and compared. However, with more than 100 clinical trials being published every week evaluating behaviour change interventions in health ^2^, the resources needed to manually maintain up-to-date evidence reviews on all the research questions of relevance to policy and practice are prohibitive. Moreover, meta-analyses of randomised trials only use a small amount of the information reported and allow only very limited conclusions to be made comparing packages of interventions in the populations and settings studied. Policymakers and practitioners need to be able to use the information to predict what will happen when interventions are delivered in the future, often in novel populations and settings. This paper reports an attempt to automate extraction of information from trial reports and also to predict interventions outcomes using features extracted from the reports. It is the first of two linked papers, the second of which developed an improved approach to predicting intervention outcomes ^3^.

Systematic reviews and meta-analyses aim to collate and synthesise evidence from studies fitting pre-specified eligibility criteria in order to estimate the effectiveness of intervention packages, such as prescription of nicotine patches to aid smoking cessation or audit and feedback to improve clinical practice ^4^. Currently these take an average of 1,000 hours of highly skilled work ^5, 6^, from pre-registration stage to publication. ‘Living’ systematic reviews are beginning to appear in the literature which avoid the problem of having to start afresh each time ^7^. However, these are also labour intensive and can only answer a limited number of questions relating to specific intervention packages versus specific comparators.

Aside from the time and resources required for evidence synthesis, the current approach cannot account adequately for the high level of context dependency in behaviour. The same intervention package may have very different effects in different populations or settings. In addition, it is rare to be able to disaggregate intervention components to assess how far particular components operate additively, synergistically or in competition. Moreover, studies are almost never completely duplicated and differences in methods can have a major impact on the findings. The result is that many systematic reviews and meta-analyses are forced to conclude that the evidence on effectiveness is mixed or weak, and heterogeneity is large. This issue cannot be resolved by more studies or reviews because the problem lies in the heterogeneity of the interventions, contexts, outcome measures and other study methods.

A possible approach to solving these problems is to use computer-based natural language processing (NLP) to extract all the key information from study reports and use machine learning (ML) to predict outcomes based on the totality of the information available. In this approach, no pre-selection needs to be made for a specific intervention package or outcome. Rather a cumulative database for a domain of interest is created with information about interventions, populations, settings and outcomes as a set of encoded features, and this database is queried to make predictions for existing or hypothetical scenarios that may vary in any of the features that have been encoded.

If all key items of information from intervention evaluation reports can be automatically extracted, this creates a knowledge base that can be queried on demand to make predictions. Such a system could provide a confidence rating depending on the extent to which there is evidence directly relevant to the query, the consistency and strength of that evidence, and show users the studies that most closely match the query and therefore have the greatest influence on the prediction.

The Human Behaviour-Change Project (HBCP) was set up in an attempt to address this need ^1, 8^. Tools such as Grobid have been developed to try to achieve this in other domains ^9, 10^. Prior work on automation of evidence synthesis includes a project by Kiritchenko *et al*. ^11^ which undertook automated extraction of 21 entities from RCTs and led to a modest time-saving as compared to a single reviewer ^12^. There is also an automated risk of bias assessment tool that encodes features of studies that may influence bias using Cochrane’s risk of bias checklist ^13^.

The HBCP aimed to develop a prototype ‘knowledge system’ that would identify BCI evaluation reports soon after publication, automatically annotate these reports to extract discrete items of information (‘entities’), and synthesise the findings to predict outcomes based on information about the intervention, population, setting and target behaviour. The HBCP was designed as a ‘proof-of-principle’ project, restricted to the exemplar case of BCIs directed at aiding smoking cessation.

**Figure 1.**
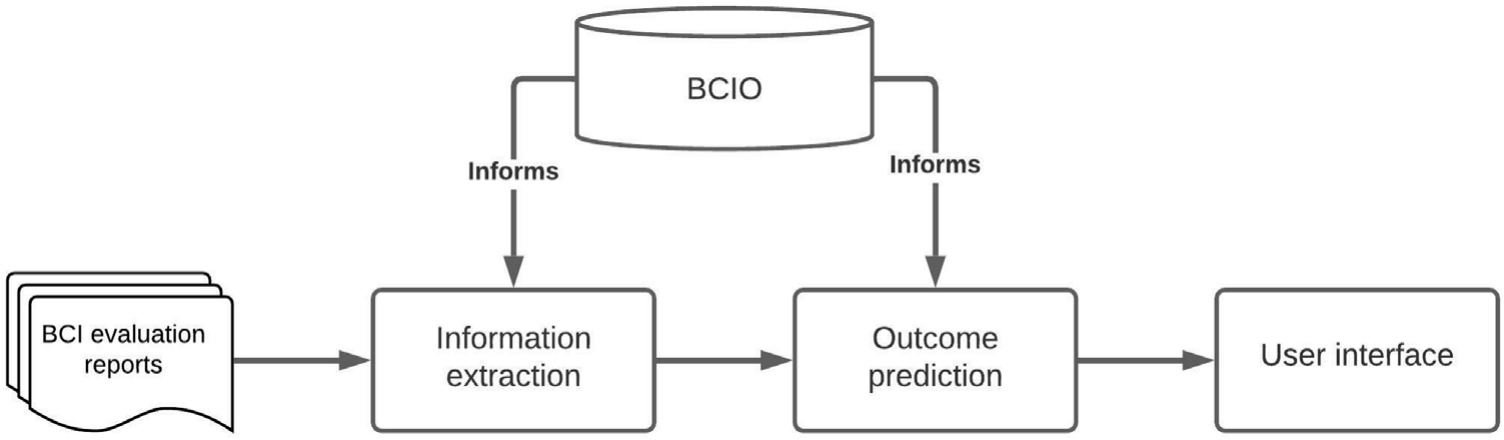
Overview of the Human Behaviour Change Project knowledge system.

Figure 1 shows how the parts of the knowledge system work together. It involves a Behaviour Change Intervention Ontology (BCIO) that delineates the items of information (entities) to be annotated, their definitions and their relationships with each other ^14^. The information extraction system used NLP to ‘read’ the study reports and create a database of ‘entities’ classified according to the ontology. The outcome prediction system used ML to create a model that allowed prediction of outcomes from information about the interventions, populations, settings and study methodology. The intention is that the ML model would be created in such a way as to be interpretable by humans, providing a basis for understanding mechanisms of action of interventions. This model could then be queried by means of a specially designed user interface.

This paper describes the development and evaluation of an initial set of information extraction and outcome prediction algorithms. It discusses the challenges faced and the lessons learnt. An evaluation of the prior stages of the knowledge system, that identify relevant research to ‘feed’ the system, is published elsewhere ^15^.

## Methods

### Overview

The information extraction and outcome prediction algorithms were developed and evaluated separately. The BCI evaluation reports used were 512 published papers reporting randomised controlled trials of smoking cessation interventions, identified from the Cochrane database of systematic reviews ^16, 17^. Entities were extracted from these reports manually and these were used to train and evaluate the information extraction algorithms. The prediction algorithms were developed and evaluated using the manually extracted entities to prevent confounding of the results with the automated information extraction evaluation.

The development and evaluation of the algorithms involved an iterative process of creating or modifying algorithms, undertaking experiments to assess their accuracy, reviewing and discussing the findings with the behavioural scientists and other team members, and repeating this process as required. Stages in this development process are published elsewhere ^18^.

### Manual information extraction

The BCIO was used to identify entities for extraction ^14^. For the purposes of this study a subset of 70 entities were identified as high priority for extraction and potential use in prediction. These were entities that appeared sufficiently frequently in study reports and were likely to be important for outcome prediction to provide a basis for training the extraction and prediction algorithms. These are listed in Appendix 4 in the *Extended data*.

Entities were divided into three types:

1. Presence-absence type: the presence of a particular entity ( *e.g.*, ‘goal-setting behaviour change technique’ in an intervention).

2. Value type: a single numerical value of a variable ( *e.g.* 26.7 for the variable ‘percentage of participants achieving 12-months of smoking abstinence’).

3. Complex type: two or more numerical values of entities linked under a parent class. ( *e.g.* 34.4 for the entity ‘percentage of White participants’, 4.5 for the entity ‘percentage of Asian participants’ under the parent class of ‘ethnic group’).

Papers were annotated manually using a coding scheme based on the BCIO to tag pieces of text in PDF documents with codes relating to entities in the ontology, using web-based EPPI-Reviewer software ^19^. For example, the phrase “44.5 years” might be annotated as characterising the ‘average age of participants’ in a study, where average age corresponds to an entity in the BCIO. As well as capturing the value of the entity, annotators also recorded the surrounding text ( *e.g.* the sentence around “44.5 years”). Where an entity related to a specific study group ( *e.g.* average of a group receiving a particular intervention in the randomised trial), it was linked to that study group (‘arm’).

Initially, two annotators independently extracted entities from the papers and discussed their annotations to resolve any discrepancies. Once acceptable inter-rater reliability had been established using the coding scheme only every fifth paper was double-coded.

### Automated information extraction

The information extraction task was treated as a form of what is termed ‘named entity recognition’ (NER) ^20^. Following a series of attempts at data extraction using different algorithms ^18, 21^, we arrived at a solution using a deep learning approach, ‘FLAIR’, an NLP framework designed to facilitate training and distribution of state-of-the-art sequence labelling, text classification and language models ^22^. At the time it was the state of the art on some standard named entity recognition ^23^ and biomedical NER tasks ^22^, using a recursive deep neural network architecture (RNN) to handle the sequential nature of words in a sentence. RNN is a bi-directional LSTM (long short-term memory) neural network ^24^ with a CRF (conditional random field) layer that ensures that labels occur in sequences ( *e.g.*, if in the sequence “University of Washington” University and Washington have same label “institution”, this model would prefer to label the entire sequence as “institution”, rather than giving single labels to each word). Appendix 2 in *Extended data* gives details of the information extraction approach.

**Figure 2.**
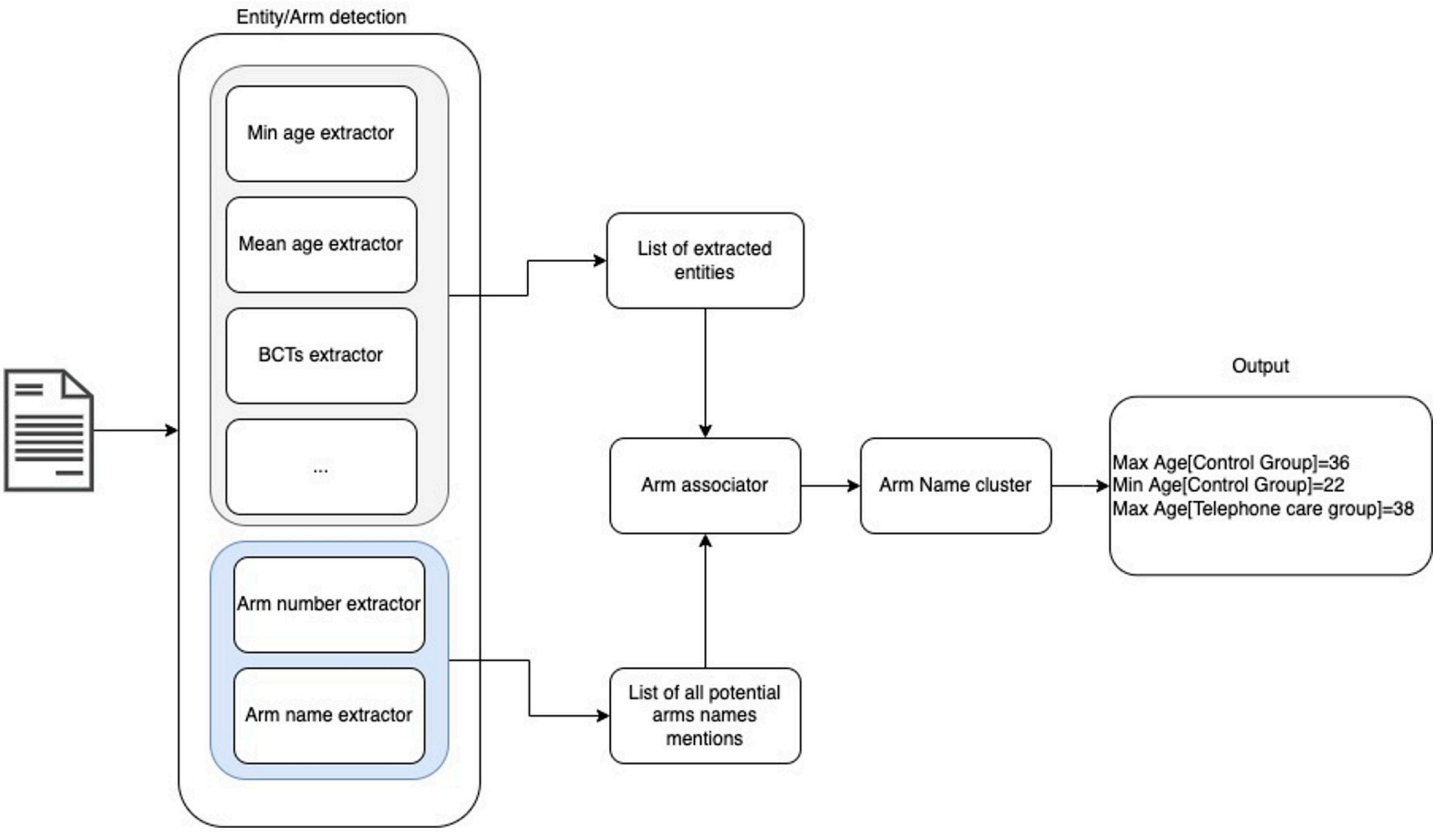
Overview of automated information extraction pipeline.

The final architecture of the information extraction algorithm is shown in Figure 2 ^25^. An NER model was trained to extract entities from reports, including the names of study arms. ‘BIO tagging’ was used for the task, where B, I and O represent the beginning, inside and outside of an entity, respectively. Our model was based on the concatenation of different embeddings, namely GloVe (pre-trained on Wikipedia and Gigaword) ^26^ and the FLAIR news-forward and news-backward contextual string embeddings (pre-trained on a 1-billion word corpus).

Much of the information to be extracted was contained in tables. To address this, the structured in table format was transformed into text by generating sentences corresponding to table content (see Appendix 2 in *Extended data* for details).

During the training stage, all sentences were extracted from PDF test documents. This was augmented with the pseudo-sentences generated from each table in the pdf. The trained model was applied to these sentences (both original and pseudo) to extract the entities.

To assign entities to study arms, a module was developed to identify, for each entity detected, the closest mention of an arm name in the text within a “window” of size t (where t was set empirically). If no instance of an arm name was found in the neighbourhood of the entity according to this algorithm, the entity was instead associated to the “whole study”.

At the end of this process, a list of tuples (arm name mention -entity) was created. However some arm name mentions would refer to the same arm, since each arm could have many mentions using different terms in an RCT report. Therefore, there was the need to cluster the arm name mentions and extract a single arm name that represented the cluster. A complete-link clustering algorithm was used where the similarity of two clusters was the similarity of their most dissimilar members. The different arm names were clustered into n classes with n corresponding to the number of arms. n was detected by exploiting the common pattern that authors often use to indicate the number of arms, *e.g.*, 'into/in' + n + groups. In the end, for each cluster, the most frequent arm name mentioned was chosen as the cluster label.

At the end of the FLAIR process, the system returned a list of all the extracted entities including arm names and associations between entities and arms where appropriate (see Figure 2).

### Outcome prediction

The manually extracted entities were used to train and evaluate the prediction algorithm. This enabled the separate evaluation of the automated information extraction and prediction algorithms. This paper reports an initial attempt to develop a prediction system. The second paper in the linked pair reports an improved approach to prediction ^3^.

Outcome prediction is a novel task in meta-analyses and meta-regressions in behavioural science. Up until now evidence synthesis has involved using forms of statistical regression to estimate differences in outcomes in existing data sets attributable to interventions or population or setting features. These are expressed as regression weights, odds ratios or similar parameters and they are derived from the data set used. Our task was to predict actual outcomes ( *e.g.*, percent achieving smoking abstinence) in unseen data sets from entities in a training set. This is a much more challenging task because it requires generalising models to new scenarios by building a model using all available data, including possibly complex causal interactions between predictors.

**Figure 3.**
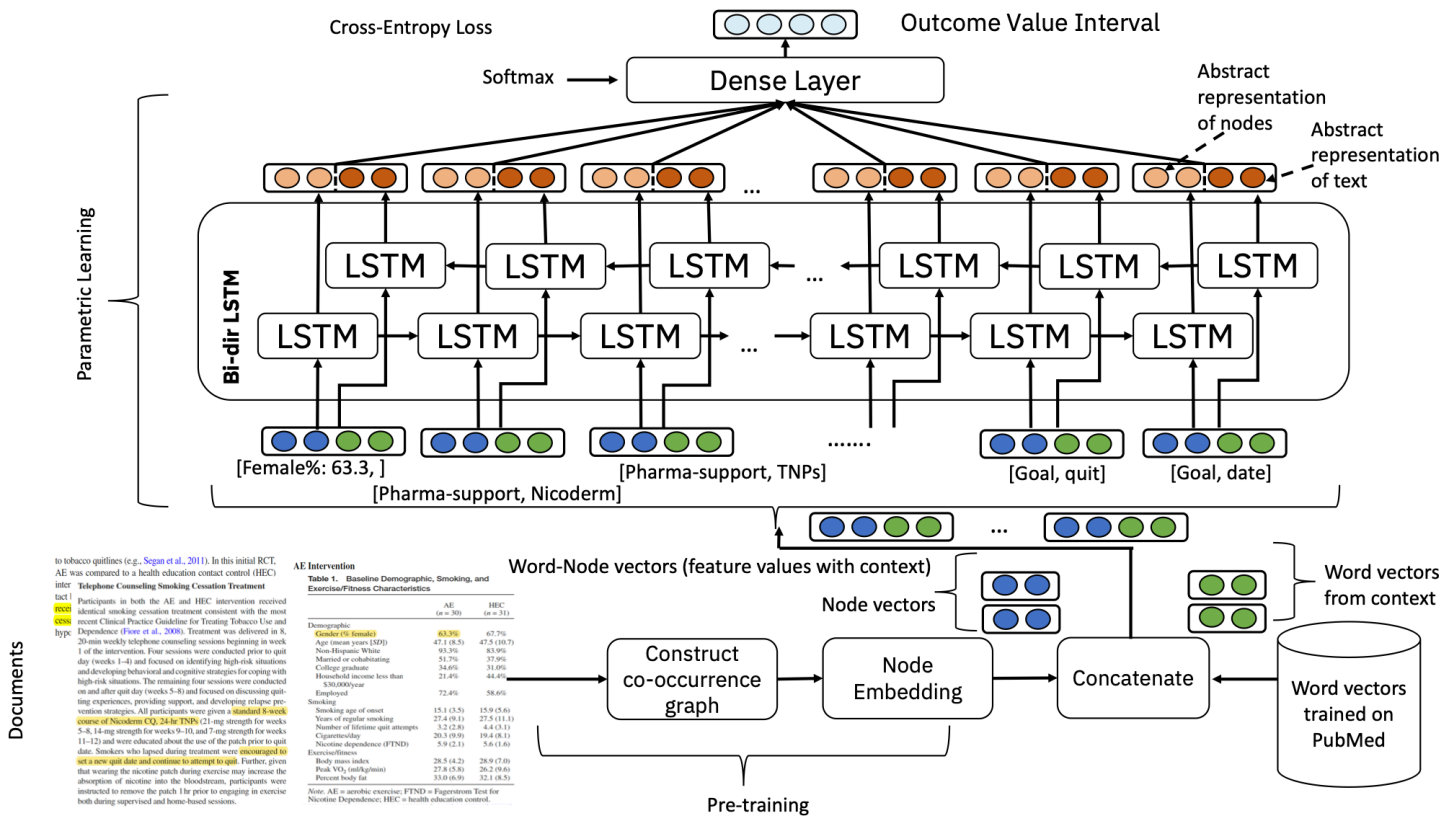
Overview of the outcome prediction ML algorithm.

To address this task, a deep learning algorithm was developed that aimed to find associations between the vectors representing all the annotated entities and the outcomes (70 in total). We used a standard deep learning sequence classification model (see Figure 3), comprised of stacked layers of LSTMs. We concatenated two different forms of embeddings, one based on a graph structure determined from co-occurrences of annotations in the corpus of annotated documents using Node2Vec ^27^, and one based on word embeddings for the features and their textual contexts using word embeddings derived from PubMed using the skip-gram algorithm. The idea behind the use of text embeddings was that the text may include predictive information that was not present in the annotations. For example, the annotation would represent all types of ‘problem solving’ for smoking cessation using a presence-absence entity called ‘problem solving’ while the text would provide further unstructured information about the type of problem solving that an ML prediction system may be able to use. Appendix 3 in *Extended data* gives details of the approach used including creation of the embeddings.

### Evaluation of information extraction and prediction algorithms

The accuracy of both the information extraction and prediction algorithms was evaluated using five-fold cross-validation, selecting 80 percent of studies to use as a training set and then attempting to predict the outcomes for the remaining 20 percent. This was repeated a further four times until all outcome values had been used once in the testing set.

The accuracy metric used for the information extraction algorithm was the ‘F1’ score, which is a standard metric for NER evaluation ^28^. This score is derived from ‘precision’ (the percentage of correct extractions, out of all occurrences for a given entity) and ‘recall’ (the percentage of correct instances, out of all actual instances of a given entity). F1 is the harmonic mean of precision and recall, which penalises being too conservative (few but very precise predictions), and being too liberal (guessing incorrectly about the presence of entities). In general, an F1 score of 0.7 is considered good but in the absence of an ability to resolve discrepancies through discussion as would be the case for a fully automated system a score of greater than 0.9 would be desirable.

As a benchmark, an F1 score was calculated for the human annotators, using the annotations from one of them (prior to discussion and agreement as to the correct annotation) as the criterion against which to compare the other.

The accuracy metric for the prediction algorithm was the root-mean-square of the error (RMSE), where error of prediction is the difference between the predicted and annotated outcome value. This is a standard metric for evaluating prediction of this kind although it tends to overweight larger inaccuracies ^29^.

The evaluation scores (RMSE) from the ML prediction system were compared with scores derived from always predicting a value that was the grand mean of the output values, *i.e.*, containing no information from the predictor entities, and a linear regression model that entered all the prediction entities together in an additive linear model.

## Results

Table 1 shows the results of the evaluation of the information extraction algorithm compared with the human benchmark. Without the opportunity for discussion, the human annotators achieved a mean F1 score of 0.76 while the automated system achieved a score of 0.42. There was considerable variability in the accuracy according to the different entities, with some of the scores for the automated algorithm being under 0.10. The scores for individual entities are given in Appendix 4 in Extended data.

**Table 1.** Results from evaliation of the information extraction algorithm.

	Automated information extraction F1 score	Human information extraction F1 score
Mean	0.42	0.76
Median	0.43	0.79
Maximum	0.88	1.00
Minimum	0.05	0.38

Only a small number of study arms were successfully identified and the algorithm used for associating the features of arms with the arm names was unsuccessful.

Table 2 shows the results of the evaluation of the outcome prediction. It is apparent that neither the attribute-only nor the combined attribute and text prediction models outperformed a prediction just based on the grand mean (but see linked paper ^3^ for an improved approach.)

**Table 2.** Results of the evaluation of the prediction algorithm.

Prediction using …	RMSE score ^1^
Grand mean	10.00
Linear regression	12.23
ML algorithm using entities only	13.92
ML algorithm using entities plus text	13.93

^1^Mean of the five 80–20 cross validation experiments

## Discussion

Developing automated ML algorithms to extract information from reports of randomised trials evaluating smoking cessation interventions proved challenging. Success was achieved at a similar level to human annotators for several types of entity, such as identifying the odds ratio of the effect size and the setting as a hospital facility (see Appendix 4 in *Extended data*) but in most cases the F1 scores were considerably lower than the human annotators. The method used to identify the study arms and associate entities with arms was not successful, and the prediction algorithm did not outperform a prediction based on the grand mean.

Several factors limited the success of the automated information extraction processes, mostly related to the way that information is presented in the reports being used, but some related to the nature of the subject matter.

1. PDFs proved problematic as a source of information. For example, different journals used very different styles and ways of handling issues such as page breaks.

2. Attempting automated information extraction from long text documents with a very large and diverse set of entities, some having a complex structure and including numeric values and units as well as classifying complex text ( *e.g.*, descriptions of behaviour change interventions), was a relatively novel task for NLP systems.

3. Reports were highly variable in the terms and phrases they used for the same entities, often using many different terms for the same entity at different points in the paper.

4. When it came to labelling the study groups or arms in the trials, authors used many different terms for the same arm within the same paper and the algorithms used were unsuccessful in using the text to identify which labels went together. Even more difficult was the task of assigning entities to study arms. This was related to the difficulties in extracting information from tables but was compounded by the huge variety of ways in which reports were structured in the way they conveyed information of this kind. Thus in some reports the only way that one could deduce that a piece of information applied to a given arm was by a heading further up in the text while in other cases information about two or more arms were included in the same sentence with use of words such as ‘respectively’ to signal which entity related to which arm.

5. Even with 512 studies to draw from, the data available to train NLP models was very sparse. For some entities, there were only tens of studies with that entity present.

6. A great deal of the information was contained in tables and converting information in tables to a form that can be used by an NLP system proved extremely challenging because of the huge variation in the way the tables in study reports are constructed. This is a known problem and advances are now being made in the conversion of tables to a form that can be used by NLP systems, but as things stand the accuracy is unlikely to be sufficient for the purposes of automated data extraction.

7. With human annotators working independently, agreement was far from perfect. This indicates the level of the challenge faced by the automated system. With human annotators it was possible to discuss and resolve any discrepancies. This was not something that could be done with an automated system.

Moreover, using an F1 score on a per-entity basis is a very lenient way of assessing performance. When the use-case on which this information extraction is based is considered, it is clear that a far higher degree of accuracy is needed than even the best-performing entities achieved. In order for the information extraction to provide data for the prediction system, it needed to associate a correctly extracted outcome with the correct arm, population, setting and intervention. Even without the challenge of identifying the correct study arm, the combined probability of the system achieving this is less than 1%.

The failure of the prediction system to outperform a model based on the grand mean of the outcome may reflect inherent difficulties in predicting outcomes rather than effect sizes in randomised trials. Thus, randomised trials are specifically designed to make study groups comparable in all respects apart from the intervention and to assess differences in outcomes observed. Predicting outcomes rather than differences in outcomes requires use of potentially large amounts of information about the populations, settings and study features.

A further issue in the case of this dataset was that outcomes in some studies were of a very different kind than outcomes in other studies. For example, if the study included a large number of people who were not smokers to start with, much higher abstinence rates were recorded than if every participant in the study started as a smoker. No prediction model would be able to cope with this without knowing about these different kinds of study.

It is also likely that prediction accuracy would have been diminished by a failure of the study reports to include crucial information about the interventions or features of the study population, setting or methodology. It has been noted using a strongly overlapping corpus of reports that intervention features are very poorly reported ^30^.

It is also possible that the particular ML approach adopted was not well suited to this particular prediction task. With such sparse data it may have overlearned associations in the training sets that were not useful when it came to prediction of outcome values in the testing sets.

The second paper in this linked series adopted a different machine learning approach, capitalising on the ontological structure of the data and using a different machine learning approach that would be more interpretable. The new approach produced much more accurate predictions ^3^.

There are a number of lessons from this work. Probably most importantly, to achieve a high level of accuracy in automated extraction of information from study reports it will be necessary for those reports to present information in a much more structured form. There is no good reason why there should be such variability in the way that information is presented, and a major effort will be needed in the coming years minimise this unnecessary variability. In many cases, this may be achieved by adopting authoring tools such as the Paper Authoring Tool (PAT) ^31^ developed as part of the HBCP, which not only ensures that information is included in study reports in a form that can easily be extracted and compared across studies, but actually generates a machine readable version of the report dramatically reducing the need for either a human or NLP system to extract the information ^32^.

Even with tools such as the PAT, studies will have unique attributes that will require natural language to describe them. However, if the reports are structured using one of these tools the NLP task will become tractable because it will always be clear to which study arm an entity belongs, and only short passages of text will need to be processed at a time to extract highly specific items of information ( *e.g.*, how a particular component of an intervention was delivered).

The ML prediction algorithm may have been hampered by sparsity of information and possibly a failure to be able to capture important predictors of outcomes, as opposed to differences in outcomes in randomised trials where study groups are comparable in all aspects apart from the intervention.

Even had the current ML approach to prediction been successful, it would not have met the requirement for providing *interpretable* predictions. A limitation of deep-learning ML systems is that they generate ‘black box’ predictions based on uninterpretable parameters in complex multi-layer models. There is a need to harness the power of ML when making predictions, but in a way that creates results that humans can understand and use to build models of behaviour. For this purpose, novel machine learning architectures are needed that are able to combine the semantic representation of domain knowledge in the form of an ontology with quantitative predictive modelling, in ways that are interpretable in terms of the ontological categories as features.

## Conclusions

An ML algorithm for extracting information from reports of randomised trials of smoking cessation interventions had limited success in achieving this goal and was unsuccessful at associating information with individual study arms. An initial ML prediction algorithm using manually extracted information from study reports did not outperform prediction using just the grand mean of outcome values, though in the linked paper a different approach was more successful ^3^. The project identified a need for much greater structure and consistency in the way that study reports convey information, including further development and adoption of tools to support researchers in creating these reports. It also identified the need for novel, semantically aware and interpretable machine learning architectures to be developed that are able to harness both domain knowledge and predictive modelling together while providing explanations in a form that leads to actionable knowledge to advance the science.

## Data Availability

Open Science Framework: Human Behaviour-Change Project. https://doi.org/10.17605/OSF.IO/UXWDB ^33^ Data are available under the terms of the Creative Commons Attribution 4.0 International license (CC-BY 4.0). Zenodo: Human Behaviour-Change Project, https://doi.org/10.5281/zenodo.8334838 ^34^ This project contains the application data and JSON files. Github: Human Behaviour-Change Project. https://github.com/HumanBehaviourChangeProject/Info-extract Data are available under the terms of the Apache License 2.0. Open Science Framework: Human Behaviour-Change Project. https://doi.org/10.17605/OSF.IO/EFP4X This project contains the following extended data: Appendix 1–4 ( https://osf.io/v8pmq) License: Creative Commons Attribution 4.0 International license (CC-BY 4.0).

## Software availability

Source code available from: https://github.com/HumanBehaviourChangeProject/Info-extract

Archived source code at time of publication: https://doi.org/10.5281/zenodo.8334838 ^34^

License: GNU General Public License v3.0

## References

[1] Michie S Thomas J Johnston M : The Human Behaviour-Change Project: harnessing the power of artificial intelligence and machine learning for evidence synthesis and interpretation. *Implement Sci.* 2017;12(1): 121. 10.1186/s13012-017-0641-5 29047393 PMC5648456

[2] West R Michie S : How many papers are published each week reporting on trials of interventions involving behavioural aspects of health?Qeios. 10.32388/U6VX2Z

[3] Hastings J Glauer M West R : Predicting outcomes of smoking cessation interventions in novel scenarios using ontology-informed, interpretable machine learning. *Wellcome Open Res.* 2023. 10.12688/wellcomeopenres.20012.2 PMC12603517 41230249

[4] Gough D Oliver S Thomas J : An Introduction to Systematic Reviews.SAGE;2017;353. Reference Source

[5] Allen IE Olkin I : Estimating Time to Conduct a Meta-analysis From Number of Citations Retrieved. *JAMA.* 1999;282(7):634–5. 10.1001/jama.282.7.634 10517715

[6] Borah R Brown AW Capers PL : Analysis of the time and workers needed to conduct systematic reviews of medical interventions using data from the PROSPERO registry. *BMJ Open.* 2017;7(2): e012545. 10.1136/bmjopen-2016-012545 28242767 PMC5337708

[7] Elliott JH Synnot A Turner T : Living systematic review: 1. Introduction—the why, what, when, and how. *J Clin Epidemiol.* 2017;91:23–30. 10.1016/j.jclinepi.2017.08.010 28912002

[8] Michie S Thomas J Mac Aonghusa P : The Human Behaviour-Change Project: An artificial intelligence system to answer questions about changing behaviour [version 1; peer review: not peer reviewed]. *Wellcome Open Res.* 2020;5:122. 10.12688/wellcomeopenres.15900.1 32566761 PMC7287511

[9] Lopez P : GROBID: Combining Automatic Bibliographic Data Recognition and Term Extraction for Scholarship Publications.In: Agosti M, Borbinha J, Kapidakis S, Papatheodorou C, Tsakonas G, editors. *Research and Advanced Technology for Digital Libraries*. Berlin, Heidelberg: Springer; (Lecture Notes in Computer Science),2009; 5714:473–4. 10.1007/978-3-642-04346-8_62

[10] Romary L Lopez P : GROBID - Information Extraction from Scientific Publications. *ERCIM News.* 2015;100. Reference Source

[11] Kiritchenko S de Bruijn B Carini S : ExaCT: automatic extraction of clinical trial characteristics from journal publications. *BMC Med Inform Decis Mak.* 2010;10(1): 56. 10.1186/1472-6947-10-56 20920176 PMC2954855

[12] Gates A Gates M Sim S : Creating efficiencies in the extraction of data from randomized trials: a prospective evaluation of a machine learning and text mining tool. *BMC Med Res Methodol.* 2021;21(1): 169. 10.23970/AHRQEPCMETHODSCREATINGEFFICIENCIES 34399684 PMC8369614

[13] Marshall IJ Kuiper J Banner E : Automating Biomedical Evidence Synthesis: RobotReviewer. *Proc Conf Assoc Comput Linguist Meet.* 2017;2017:7–12. 10.18653/v1/P17-4002 29093610 PMC5662138

[14] Michie S West R Finnerty AN : Representation of behaviour change interventions and their evaluation: Development of the Upper Level of the Behaviour Change Intervention Ontology [version 2; peer review: 2 approved]. *Wellcome Open Res.* 2020;5: 123. 10.12688/wellcomeopenres.15902.2 33614976 PMC7868854

[15] Shemilt I Arno A Thomas J : Cost-effectiveness of Microsoft Academic Graph with machine learning for automated study identification in a living map of coronavirus disease 2019 (COVID-19) research [version 1; peer review: 2 approved with reservations]. *Wellcome Open Res.* 2021;6:210. 10.12688/wellcomeopenres.17141.1 38686019 PMC11056680

[16] Livingstone-Banks J Lindson N Hartmann-Boyce J : Effects of interventions to combat tobacco addiction: Cochrane update of 2019 and 2020 reviews. *Addiction.* 2022;117(6):1573–88. 10.1111/add.15769 34859525

[17] Starr M Chalmers I Clarke M : The origins, evolution, and future of *The Cochrane Database of Systematic Reviews*. *Int J Technol Assess Health Care.* 2009;25(Suppl 1):182–95. 10.1017/S026646230909062X 19534840

[18] Ganguly D Hou Y Deleris LA : Information Extraction of Behavior Change Intervention Descriptions. *AMIA Jt Summits Transl Sci Proc.* 2019;2019:182–91. 31258970 PMC6568066

[19] Thomas J Graziosi S Brunton J : EPPI-Reviewer: advanced software for systematic reviews, maps and evidence synthesis.EPPI-Centre Software. London: UCL Social Research Institute;2020.

[20] Nadeau D Sekine S : A survey of named entity recognition and classification. *Lingvisticæ Investigationes.* 2007;30(1):3–26. 10.1075/li.30.1.03nad

[21] Ganguly D Deleris LA Deleris PM : Unsupervised Information Extraction from Behaviour Change Literature. *Stud Health Technol Inform.* 2018;247:680–684. 29678047

[22] Akbik A Bergmann T Vollgraf R : Pooled Contextualized Embeddings for Named Entity Recognition. In: *Proceedings of the 2019 Conference of the North American Chapter of the Association for Computational Linguistics: Human Language Technologies, Volume 1 (Long and Short Papers)*. Minneapolis, Minnesota: Association for Computational Linguistics;2019;724–8. [cited 2023 Jan 30]. 10.18653/v1/N19-1078

[23] Aramaki E Miura Y Tonoike M : TEXT2TABLE: medical text summarization system based on named entity recognition and modality identification. In: *Proceedings of the Workshop on BioNLP - BioNLP ’ 09*. Boulder, Colorado: Association for Computational Linguistics;2009;185. [cited 2023 Jan 30]. Reference Source

[24] Hochreiter S Schmidhuber J : Long Short-Term Memory. *Neural Comput.* 1997;9(8):1735–80. 10.1162/neco.1997.9.8.1735 9377276

[25] Bonin F Gleize M Hou Y : Knowledge Extraction and Prediction from Behavior Science Randomized Controlled Trials: A Case Study in Smoking Cessation. *AMIA Annu Symp Proc.* 2021;2020:253–62. 33936397 PMC8075460

[26] Pennington J Socher R Manning C : Glove: Global Vectors for Word Representation. In: *Proceedings of the 2014 Conference on Empirical Methods in Natural Language Processing (EMNLP)*. Doha, Qatar: Association for Computational Linguistics;2014;1532–43. [cited 2023 Jan 30]. 10.3115/v1/D14-1162

[27] Grover A Leskovec J : node2vec: Scalable Feature Learning for Networks. In: *Proceedings of the 22nd ACM SIGKDD International Conference on Knowledge Discovery and Data Mining*. New York, NY, USA: Association for Computing Machinery;2016;855–64. (KDD ’ 16). [cited 2023 Jan 18]. 10.1145/2939672.2939754 PMC5108654 27853626

[28] Yedidia AB : Against the F-score.2016; [cited 2023 Jan 30]. Reference Source

[29] Chai T Draxler RR : Root mean square error (RMSE) or mean absolute error (MAE)? - Arguments against avoiding RMSE in the literature. *Geosci Model Dev.* 2014;7(3):1247–50. 10.5194/gmd-7-1247-2014

[30] de Bruin M Black N Javornik N : Underreporting of the active content of behavioural interventions: A systematic review and meta-analysis of randomised trials of smoking cessation interventions. *Health Psychol Rev.* 2021;15(2):195–213. 10.1080/17437199.2019.1709098 31906781

[31] West R : An online Paper Authoring Tool (PAT) to improve reporting of, and synthesis of evidence from, trials in behavioral sciences. *Health Psychol.* 2020;39(9):846–850. 10.1037/hea0000927 32833486

[32] West R : Addiction paper authoring tool (PAT): a guide. Qeios,2020;10: L2KF6W. 10.32388/L2KF6W

[33] West R Michie S Shawe-Taylor J : Human Behaviour-Change Project. 2020. 10.17605/OSF.IO/UXWDB PMC7287511 32566761

[34] Bonin F : Using machine learning to extract information and predict outcomes from reports of randomised trials of smoking cessation interventions in the Human Behaviour-Change Project - Source Code. 2023. 10.5281/zenodo.8334838 PMC11109593 38779058

