# Supplementary material for: Using machine learning to extract information and predict outcomes from reports of randomised trials of smoking cessation interventions in the Human Behaviour-Change Project
Source: Wellcome Open Res. 2024 Nov 20;8:452. Originally published 2023 Oct 12. [Version 2] doi: 10.12688/wellcomeopenres.20000.2 (PMC11109593; doi:10.12688/wellcomeopenres.20000.2)
Supplement: Peer review history

# Reviewer report: Reviewer response for version 2

**DOI:** 10.21956/wellcomeopenres.25824.r113600

**Published:** 2025-01-06

**Authors:** Kate Frazer

**Affiliations:** 1 University College Dublin, Dublin, Leinster, Ireland

Thank you for asking me to review this paper. It is an important paper that presents the challenges of using an ML-designed algorithm to extract information from reports of randomised trials of smoking cessation interventions. The paper also highlights the discrepancies in RCT reporting that influence ML and presents the need for using PAT or similar reporting tools for consistency.

Is the work clearly and accurately presented and does it cite the current literature?

Yes

If applicable, is the statistical analysis and its interpretation appropriate?

Yes

Are all the source data underlying the results available to ensure full reproducibility?

Yes

Is the study design appropriate and is the work technically sound?

Yes

Are the conclusions drawn adequately supported by the results?

Yes

Are sufficient details of methods and analysis provided to allow replication by others?

Yes

Reviewer Expertise:

Associate Professor and Cochrane trained author and expert in the research areas of systematic reviews, tobacco control, patient and public involvement, and inclusion health.

I confirm that I have read this submission and believe that I have an appropriate level of expertise to confirm that it is of an acceptable scientific standard.

# Reviewer report: Reviewer response for version 1

**DOI:** 10.21956/wellcomeopenres.22146.r68632

**Published:** 2024-05-11

**Authors:** John Beverley

**Affiliations:** 1 Department of Philosophy, University at Buffalo, Buffalo, New York, USA

**Summary**

 This study aimed to develop and evaluate machine learning (ML) algorithms for extracting information from randomized trial reports of smoking cessation interventions and predicting outcomes. 70 items across 512 reports were annotated using the Behaviour Change Intervention Ontology. The results were used to train ML algorithms for automatic extraction. A FLAIR-based extraction strategy resulted in an average F1 score of 0.42, which was significantly lower than that of human annotators: 0.75. Moreover, the ML outcome prediction algorithm did not surpass basic prediction models. The authors highlight strategies for improving these results, one of which is pursued in a separate research article using an ontology to improve the ML algorithm.

**General Remarks**

 I appreciate that this article aims to empirically explore information extraction and outcome prediction strategies with an eye towards comparing these results against strategies employing ontologies to inform algorithm development. Such comparative work is sorely needed to determine more precisely where traditional ML strategies may be supplemented by ontology and perhaps knowledge graph techniques.

 That said, this article reads as the first part of a longer article, the second part being the follow-up study employing ontologies to inform ML. This is perhaps due to there being, to my mind, significant room to expand this article. In particular, the authors place in the appendix detailed descriptions of the FLAIR strategy pursued and decisions made to address issues encountered during training. Moving this information to the body of the article would, I think, be an improvement.

 Similarly, expanding on how exactly human annotators performed their tasks, how they resolved disagreements, and what ‘acceptable inter-rater reliability’ consists in, would result in this article feeling more of a stand-alone piece.

**Further Suggestions**

 The authors gloss over the ‘average’ number of hours it takes to conduct meta-analyses and systematic reviews too quickly, citing two articles in support. The JAMA Research Letter cited suggests the number of hours taken is a function of the number of citations in a given analysis while the ‘1000’ hours figure is from a paper cited by the Research Letter but which is focused on ovarian cancer literature. The second article cited actually claims the JAMA Research article results are too simplistic and suggest more hours are needed.

 It is hard to evaluate the suggested concerns with ‘living’ systematic reviews because the authors do not say what those concerns are.

 The authors write: “The same intervention package may have very different effects in different populations or settings. In addition, it is rare to be able to disaggregate intervention components to assess how far particular components operate additively, synergistically or in competition. Moreover, studies are almost never completely duplicated and differences in methods can have a major impact on the findings. The result is that many systematic reviews and meta-analyses are forced to conclude that the evidence on effectiveness is mixed or weak, and heterogeneity is large.”

 The authors then suggest creating an NLP-generated knowledge base that can be queried might solve these problems. However, other than gesturing at “confidence rating” and “evidence” possibly provided by such a workflow, it is unclear how such a strategy could address the significant issues raise. My suspicion is that even with the knowledge base, one will still only be able to “conclude that the evidence on effectiveness is mixed or weak, and heterogeneity is large.”

 Related, the authors write: “Such a system could provide a confidence rating depending on the extent to which there is evidence directly relevant to the query…” It is, however, unclear how the authors understand ‘relevant to the query’? I encourage expanding on this remark since seems part of the fundamental motivation for the present research.

 The authors write: “If all key items of information from intervention evaluation reports can be automatically extracted, this creates a knowledge base…” Automatically extracting information does not necessarily result in a knowledge base that can be queried. I invite you to be more careful about the intended workflow and its results, even when described in passing.

 The authors write: “The Human Behaviour-Change Project (HBCP) was set up in an attempt to address this need.” Please state carefully what need you mean here.

 The authors write: “The intention is that the ML model would be created in such a way as to be interpretable by humans, providing a basis for understanding mechanisms of action of interventions.” I assume this owes to the use of BCIO in ML model development; if so, I would encourage the authors to state more clearly how such a strategy promotes interpretability. If not, then I encourage the authors explain what they mean.

 The authors write: “These were entities that appeared sufficiently frequently in study reports and were likely to be important for outcome prediction to provide a basis for training the extraction and prediction algorithms.” Perhaps I misunderstand, but it in the appendix the authors describe needing to employ an ‘upsampling’ for text strings lacking a sufficient number of BCIO annotations. I understand that what counts as sufficient representation in study reports may not count as sufficient for training a model. This does, however, raise the question of how the authors understand “sufficiently frequently” with respect to representation in study reports and with respect to training a model.

Is the work clearly and accurately presented and does it cite the current literature?

Partly

If applicable, is the statistical analysis and its interpretation appropriate?

Yes

Are all the source data underlying the results available to ensure full reproducibility?

Partly

Is the study design appropriate and is the work technically sound?

Yes

Are the conclusions drawn adequately supported by the results?

Yes

Are sufficient details of methods and analysis provided to allow replication by others?

Partly

Reviewer Expertise:

Leveraging ontologies and knowledge graphs in support of ML strategies.

I confirm that I have read this submission and believe that I have an appropriate level of expertise to confirm that it is of an acceptable scientific standard, however I have significant reservations, as outlined above.

# Response

**Published:** 2024-11-13

**Authors:** Paulina Margarete Schenk

**Affiliations:** University College London, London, England, UK

**Summary**

 This study aimed to develop and evaluate machine learning (ML) algorithms for extracting information from randomized trial reports of smoking cessation interventions and predicting outcomes. 70 items across 512 reports were annotated using the Behaviour Change Intervention Ontology. The results were used to train ML algorithms for automatic extraction. A FLAIR-based extraction strategy resulted in an average F1 score of 0.42, which was significantly lower than that of human annotators: 0.75. Moreover, the ML outcome prediction algorithm did not surpass basic prediction models. The authors highlight strategies for improving these results, one of which is pursued in a separate research article using an ontology to improve the ML algorithm.

**General Remarks**

 I appreciate that this article aims to empirically explore information extraction and outcome prediction strategies with an eye towards comparing these results against strategies employing ontologies to inform algorithm development. Such comparative work is sorely needed to determine more precisely where traditional ML strategies may be supplemented by ontology and perhaps knowledge graph techniques.

 That said, this article reads as the first part of a longer article, the second part being the follow-up study employing ontologies to inform ML. This is perhaps due to there being, to my mind, significant room to expand this article. In particular, the authors place in the appendix detailed descriptions of the FLAIR strategy pursued and decisions made to address issues encountered during training. Moving this information to the body of the article would, I think, be an improvement.

***Response:*** *This article already contained a lot of information and reported the findings of the first phase of the research. It was more natural and made digesting the information easier for the readers to have the second phase in a separate article that we refer to. Following extensive discussion in the team it was decided that the technical details were better placed in an Appendix to maintain the narrative flow for non-technical readers.*

 Similarly, expanding on how exactly human annotators performed their tasks, how they resolved disagreements, and what ‘acceptable inter-rater reliability’ consists in, would result in this article feeling more of a stand-alone piece.

***Response:*** *Information on this is now included, together with a reference to a more complete description.*

**Further Suggestions**

 The authors gloss over the ‘average’ number of hours it takes to conduct meta-analyses and systematic reviews too quickly, citing two articles in support. The JAMA Research Letter cited suggests the number of hours taken is a function of the number of citations in a given analysis while the ‘1000’ hours figure is from a paper cited by the Research Letter but which is focused on ovarian cancer literature. The second article cited actually claims the JAMA Research article results are too simplistic and suggest more hours are needed.

***Response:*** *We agree that the number of hours varies but the point being made is uncontested – that is a very large number.*

 It is hard to evaluate the suggested concerns with ‘living’ systematic reviews because the authors do not say what those concerns are.

***Response:*** *In fact we do say: “Living’ systematic reviews are beginning to appear in the literature which avoid the problem of having to start afresh each time. However, these are also labour intensive and can only answer a limited number of questions relating to specific intervention packages versus specific comparators.”*

 The authors write: “The same intervention package may have very different effects in different populations or settings. In addition, it is rare to be able to disaggregate intervention components to assess how far particular components operate additively, synergistically or in competition. Moreover, studies are almost never completely duplicated and differences in methods can have a major impact on the findings. The result is that many systematic reviews and meta-analyses are forced to conclude that the evidence on effectiveness is mixed or weak, and heterogeneity is large.” The authors then suggest creating an NLP-generated knowledge base that can be queried might solve these problems. However, other than gesturing at “confidence rating” and “evidence” possibly provided by such a workflow, it is unclear how such a strategy could address the significant issues raise. My suspicion is that even with the knowledge base, one will still only be able to “conclude that the evidence on effectiveness is mixed or weak, and heterogeneity is large.”

***Response:*** *We disagree with this pessimistic opinion which appears to imply that there is no point in attempting to be more precise and accurate when integrating evidence and making predictions. There will obviously be limits to how far we can go in terms of understanding complex causal interactions, but the further we go the better we will be at developing effective interventions.*

 Related, the authors write: “Such a system could provide a confidence rating depending on the extent to which there is evidence directly relevant to the query…” It is, however, unclear how the authors understand ‘relevant to the query’? I encourage expanding on this remark since seems part of the fundamental motivation for the present research.

***Response:*** *The extent to which evidence is relevant to a query will depend on the degree of match in terms of the behaviour, population and setting. Thus, if the query is about how to improve attendance at annual general health checks in a district in Japan, evidence will be more or less relevant depending on how far it involved health checks of that kind versus, say cancer screening, features of the population and its culture, and features of settings in which the intervention and behaviour were assessed. We have included this in the text now.*

 The authors write: “If all key items of information from intervention evaluation reports can be automatically extracted, this creates a knowledge base…” Automatically extracting information does not necessarily result in a knowledge base that can be queried. I invite you to be more careful about the intended workflow and its results, even when described in passing. ***Response:*** *We have changed the text to read: “If all key items of information from intervention evaluation reports can be automatically extracted and stored, this creates the foundation of a knowledge base that can be queried on demand to make predictions.”*

 The authors write: “The Human Behaviour-Change Project (HBCP) was set up in an attempt to address this need.” Please state carefully what need you mean here.

***Response:*** *We believe that what ‘this need’ refers to is clear already since it is stated in the preceding text.*

 The authors write: “The intention is that the ML model would be created in such a way as to be interpretable by humans, providing a basis for understanding mechanisms of action of interventions.” I assume this owes to the use of BCIO in ML model development; if so, I would encourage the authors to state more clearly how such a strategy promotes interpretability. If not, then I encourage the authors explain what they mean.

***Response:*** *We have added text to address this: “The ML model learned rules involving combinations of the presence versus absence of BCIO entities that predict meaningful increases or decreases in outcome, estimating the size of this increase or decrease.”*   This statement captures an objective for the design of the system, which  could be realised in different ways depending on different ML algorithms and approaches. Indeed, the objective would be that the BCIO would serve as the language in which data is organised and that at the same time it would  serve as the language in which the interpretability approaches give their results. However, this is not currently possible for many of the ML approaches that are currently available. See our linked paper on the ontology-informed ML approach that we developed subsequently to the work reported in this paper, specifically to address this requirement.

 The authors write: “These were entities that appeared sufficiently frequently in study reports and were likely to be important for outcome prediction to provide a basis for training the extraction and prediction algorithms.” Perhaps I misunderstand, but it in the appendix the authors describe needing to employ an ‘upsampling’ for text strings lacking a sufficient number of BCIO annotations. I understand that what counts as sufficient representation in study reports may not count as sufficient for training a model. This does, however, raise the question of how the authors understand “sufficiently frequently” with respect to representation in study reports and with respect to training a model.

***Response:*** *We made a pragmatic choice. The corpus, while large in systematic review terms, was small when it comes to training machine learning models. In practice wherever we set the threshold for number of instances, it was possible that we might get better performance with more to work with. One technique for approximating this is upsampling. In the event it was not successful, but it was important to report what we had done and what we found.*

# Reviewer report: Reviewer response for version 1

**DOI:** 10.21956/wellcomeopenres.22146.r74559

**Published:** 2024-05-11

**Authors:** Sheleigh Lawler, Hollie Bendotti

**Affiliations:** 1 University of Queensland, Herston, Queensland, Australia

This study reports the development and evaluation of a machine learning algorithm for extracting information from published trials to predict outcomes of behaviour change interventions. Using reports of smoking cessation interventions human annotated entities, the paper describes results of the algorithm training and its ability to automatically extract the human annotated information. The manuscript is very interesting, novel and clearly written with appropriate discussion points and conclusion. While we are not experts in NLP frameworks and algorithms, we commend the authors for their clear explanation of methods and processes used. This paper demonstrates the complexity of human research and that trying to bring simplicity to that with AI is not a full proof solution.

 Many of my original comments/thoughts were able to answered by the clear discussion points/limitations but I have some minor comments:

- Does the HBCP knowledge system have the capacity to scan supplementary files etc as many trials report more detailed information in these files due to journal constraints?
- Would solely using the BCIO to train the model present a possible risk to the algorithms reliability? There is much nuance in descriptions and delivery of behaviour change interventions. Similarly, which was touched in the discussion, relying on the binary of presence/absence of entities presents risk in missing information as they trials are not reported in a consistent or systematic way. How would a predictive algorithm alone account for this nuance and heterogeneity in reporting of data?
- We would recommend including inter-rater reliability scores of manual annotation. How many people completed this?
- Typo on Table 1 title – ‘evaluation’
- The difference between precision and recall wasn’t entirely clear. We would recommend further explaining the difference between instances and extractions.
- Why did some smoking cessation trials used in the training set include large numbers of people who don’t smoke?

Is the work clearly and accurately presented and does it cite the current literature?

Yes

If applicable, is the statistical analysis and its interpretation appropriate?

Yes

Are all the source data underlying the results available to ensure full reproducibility?

Yes

Is the study design appropriate and is the work technically sound?

Yes

Are the conclusions drawn adequately supported by the results?

Yes

Are sufficient details of methods and analysis provided to allow replication by others?

Yes

Reviewer Expertise:

Public Health, behavioural science

We confirm that we have read this submission and believe that we have an appropriate level of expertise to confirm that it is of an acceptable scientific standard.

# Response

**Published:** 2024-11-13

**Authors:** Paulina Margarete Schenk

**Affiliations:** University College London, London, England, UK

This study reports the development and evaluation of a machine learning algorithm for extracting information from published trials to predict outcomes of behaviour change interventions. Using reports of smoking cessation interventions human annotated entities, the paper describes results of the algorithm training and its ability to automatically extract the human annotated information. The manuscript is very interesting, novel and clearly written with appropriate discussion points and conclusion. While we are not experts in NLP frameworks and algorithms, we commend the authors for their clear explanation of methods and processes used. This paper demonstrates the complexity of human research and that trying to bring simplicity to that with AI is not a full proof solution.

 Many of my original comments/thoughts were able to answered by the clear discussion points/limitations but I have some minor comments:

- Does the HBCP knowledge system have the capacity to scan supplementary files etc as many trials report more detailed information in these files due to journal constraints?
***Response:*** *We know of work that attempts to link multiple reports of the same study, and also reports of a study with a trials registry record. It would be possible to integrate this into the system, though did not have capacity to do this in the prototype system.*  
- Would solely using the BCIO to train the model present a possible risk to the algorithms reliability? There is much nuance in descriptions and delivery of behaviour change interventions. Similarly, which was touched in the discussion, relying on the binary of presence/absence of entities presents risk in missing information as they trials are not reported in a consistent or systematic way. How would a predictive algorithm alone account for this nuance and heterogeneity in reporting of data?
***Response:*** *The BCIO is designed to classify entities at a level of specificity that allows meaningful patterns of causal association with outcomes to be established. This is always a pragmatic choice. In principle one can often be more specific but at a cost of fewer examples from which to detect patterns. The semantic hierarchy of the BCIO allows for increased specificity to be captured as and when data become available, while preserving the opportunity to observe patterns among the more general classes. *  
- We would recommend including inter-rater reliability scores of manual annotation. How many people completed this?
***Response:*** *See above.*  
- Typo on Table 1 title – ‘evaluation’
***Response:*** *This has now been corrected.*  
- The difference between precision and recall wasn’t entirely clear. We would recommend further explaining the difference between instances and extractions.
***Response:*** *See above*  
- Why did some smoking cessation trials used in the training set include large numbers of people who don’t smoke?
***Response:*** *These were relapse prevention trials. We have added this explanation.*

# Reviewer report: Reviewer response for version 1

**DOI:** 10.21956/wellcomeopenres.22146.r72790

**Published:** 2024-02-29

**Authors:** Justin White

**Affiliations:** 1 Department of Health Law, Policy and Management, Boston University, Boston, Massachusetts, USA

This study evaluates a novel approach to natural language processing for the extraction of key information from study reports and then uses a machine learning algorithm to predict outcomes based on certain variables included in the extracted information. The authors apply this approach to RCTs of smoking cessation interventions as part of the Human Behaviour-Change Project. I speak from the perspective of a researcher who has led multiple RCTs of behavioral interventions for smoking cessation and conducted a systematic review and meta-analysis of a particular behavioral intervention for smoking cessation. I am not qualified to assess the validity of the extraction and machine learning algorithms used by the authors. Each aspect of the study—the data extraction and the prediction—is innovative and highly significant for research. The ability to extract information algorithmically from published studies would be tremendously valuable for evidence syntheses, saving countless hours of person time and facilitating `living’ systematic reviews. Using extracted information to predict which features of the interventions, populations, settings, and outcomes are most effective is an ambitious task but no less valuable. This could be used for evaluating the quality of the evidence base and as an input into selecting the features of interventions to test prospectively.

 The study found that the extraction algorithm failed to match the accuracy of human annotators and failed to reach a reliable level of accuracy, as measured by an F1 score. The prediction algorithm failed to outperform a prediction based on the grand mean of the algorithm output values or a linear regression that entered all the output values. Nevertheless, the attempt provides some valuable lessons for future research, and the authors identify several important reasons why this attempt came up short. In a companion paper, the authors build on this work to test a different machine learning approach that succeeded in predicting outcomes in smoking cessation interventions with moderate accuracy.  In sum, the authors have tackled an important challenge, and produced findings that, while null, still have significance for the fields of evidence synthesis, natural language processing and machine learning, and behavior change interventions. 

 Overall, I found the paper to be highly readable given the complexity of the methodology. I only had a handful of minor comments that do not need to be addressed to make the paper scientifically sound.

  

1. Does the data extraction model attempt to impute missing data about entities? I believe it does not, although it would be helpful to state explicitly how the extraction algorithm handled missing entity data. Further, I wonder if one promising use of ML for data extraction could be to impute missing entity data for use in meta-analyses. If the authors agree, this might be worth commenting on.
2. What was the inter-rater reliability for the manual coding procedure? I would report it.
3. The title and table note for Appendix 4 do not make clear that the table contains a list of the 70 extracted entities. Moreover, the word “entities” does not appear in Appendix 4. This could be clarified. Some of the entities listed under Attribute, notably the ones with BCIO of “Mode of delivery,” have vague descriptions. For example, what does “distance” represent? I suggest providing clearer descriptions for attributes.
4. I did not understand the distinction between precision and recall as described in the calculation of the F1 score. What is the difference between the percentage of correct extractions and the percentage of correct instances?

Is the work clearly and accurately presented and does it cite the current literature?

Yes

If applicable, is the statistical analysis and its interpretation appropriate?

I cannot comment. A qualified statistician is required.

Are all the source data underlying the results available to ensure full reproducibility?

Yes

Is the study design appropriate and is the work technically sound?

Yes

Are the conclusions drawn adequately supported by the results?

Yes

Are sufficient details of methods and analysis provided to allow replication by others?

Yes

Reviewer Expertise:

Smoking cessation interventions, clinical trials

I confirm that I have read this submission and believe that I have an appropriate level of expertise to confirm that it is of an acceptable scientific standard.

# Response

**Published:** 2024-11-13

**Authors:** Paulina Margarete Schenk

**Affiliations:** University College London, London, England, UK

This study evaluates a novel approach to natural language processing for the extraction of key information from study reports and then uses a machine learning algorithm to predict outcomes based on certain variables included in the extracted information. The authors apply this approach to RCTs of smoking cessation interventions as part of the Human Behaviour-Change Project. I speak from the perspective of a researcher who has led multiple RCTs of behavioral interventions for smoking cessation and conducted a systematic review and meta-analysis of a particular behavioral intervention for smoking cessation. I am not qualified to assess the validity of the extraction and machine learning algorithms used by the authors. Each aspect of the study—the data extraction and the prediction—is innovative and highly significant for research. The ability to extract information algorithmically from published studies would be tremendously valuable for evidence syntheses, saving countless hours of person time and facilitating `living’ systematic reviews. Using extracted information to predict which features of the interventions, populations, settings, and outcomes are most effective is an ambitious task but no less valuable. This could be used for evaluating the quality of the evidence base and as an input into selecting the features of interventions to test prospectively.

 The study found that the extraction algorithm failed to match the accuracy of human annotators and failed to reach a reliable level of accuracy, as measured by an F1 score. The prediction algorithm failed to outperform a prediction based on the grand mean of the algorithm output values or a linear regression that entered all the output values. Nevertheless, the attempt provides some valuable lessons for future research, and the authors identify several important reasons why this attempt came up short. In a companion paper, the authors build on this work to test a different machine learning approach that succeeded in predicting outcomes in smoking cessation interventions with moderate accuracy.  In sum, the authors have tackled an important challenge, and produced findings that, while null, still have significance for the fields of evidence synthesis, natural language processing and machine learning, and behavior change interventions. 

 Overall, I found the paper to be highly readable given the complexity of the methodology. I only had a handful of minor comments that do not need to be addressed to make the paper scientifically sound.

  

1. Does the data extraction model attempt to impute missing data about entities? I believe it does not, although it would be helpful to state explicitly how the extraction algorithm handled missing entity data. Further, I wonder if one promising use of ML for data extraction could be to impute missing entity data for use in meta-analyses. If the authors agree, this might be worth commenting on. ***Response:*** *It does not aim to impute missing data. It’s possible this might be a good idea in the future, but current technology struggles to reach reasonable levels of accuracy even without trying to impute, so we felt it was important to focus on accurate data extraction first.*  
2. What was the inter-rater reliability for the manual coding procedure? I would report it.
***Response:*** *The krippendorf’s alpha as an index of overall inter-rater reliability for a test set of 80 reports was 0.74. This has been added to the paper together with a citation.*  
3. The title and table note for Appendix 4 do not make clear that the table contains a list of the 70 extracted entities. Moreover, the word “entities” does not appear in Appendix 4. This could be clarified. Some of the entities listed under Attribute, notably the ones with BCIO of “Mode of delivery,” have vague descriptions. For example, what does “distance” represent? I suggest providing clearer descriptions for attributes.
***Response:*** *We have added the clarification*s.  
4. I did not understand the distinction between precision and recall as described in the calculation of the F1 score. What is the difference between the percentage of correct extractions and the percentage of correct instances?
***Response:*** *We have updated the section on precision and recall as follows: “‘… precision’ (the percentage of correct instances, out of all retrieved instances for a given entity) and ‘recall’ (the percentage of correct instances, out of all actual instances of a given entity whether or not they were retrieved)….”*
